# Effects of Three Feed Additives on the Culturable Microbiota Composition and Histology of the Anterior and Posterior Intestines of Zebrafish (*Danio rerio*)

**DOI:** 10.3390/ani12182424

**Published:** 2022-09-14

**Authors:** Alexei Nikiforov-Nikishin, Svetlana Smorodinskaya, Nikita Kochetkov, Dmitry Nikiforov-Nikishin, Valery Danilenko, Oleg Bugaev, Aleksey Vatlin, Nina Abrosimova, Sergei Antipov, Alexander Kudryavtsev, Viktor Klimov

**Affiliations:** 1 Faculty of Biotechnology and Fisheries, Moscow State University of Technologies and Management (FCU), 73 Zemlyanoy Val Str., 109004 Moscow, Russia; 2 Laboratory of Bacterial Genetics, Vavilov Institute of General Genetics, Russian Academy of Sciences, 119333 Moscow, Russia; 3 Department of Aquaculture Techniques, Don State Technical University, Gagarin Square 1, 344003 Rostov-on-Don, Russia; 4 Department of Biophysics and Biotechnology, Voronezh State University, University Square 1, 394063 Voronezh, Russia; 5 Engelhardt Institute of Molecular Biology, Russian Academy of Sciences, 119991 Moscow, Russia

**Keywords:** intestinal microbiota, trace elements, butyric acid, lycopene, cultural-dependent method, intestinal histology

## Abstract

**Simple Summary:**

This work is dedicated to the study of the effects of three feed additives that are promising for use in aquaculture on the culturable microbial community and the tissue structure (histology) of the intestines of laboratory zebrafish. The feed additives used in this research have different functional properties: (i) micronutrient chelated compounds, which eliminate mineral nutrition deficiencies; (ii) butyric acid, a postbiotic drug aimed at stimulating intestinal bacteria growth and improving immunity; and (iii) lycopene, a carotenoid and wide-spectrum antioxidant. All of the tested supplements resulted in changes in the intestinal microbial community in both the anterior and posterior intestines. The change in gut microbiota correlated with histological changes in the intestines. The results of the study showed that in most concentrations, the feed additives had a positive effect on certain groups of microorganisms. The method of microbiota research based on microorganism cultivation used in this work makes it possible to evaluate the effect of feed additives on the relative abundance of key representatives of the microbial community.

**Abstract:**

In this study, the effect of three promising feed additives (chelated compounds of trace elements, butyric acid, lycopene) on changes in the culturable microbiota and histological parameters of two sections of the intestines of *Danio rerio* (zebrafish) was studied. The use of these feed additives can help to eliminate the deficiency of trace elements, modulate the composition of the microbiota due to the postbiotic properties of butyric acid, and reduce oxidative stress when using lycopene. Incorporation of the investigated supplements in the feed resulted in a significant change in the relative abundance of certain groups of microorganisms. The taxonomic diversity of cultured microorganisms did not differ in the anterior and posterior intestines, while there were differences in the relative abundance of these microorganisms. The most sensitive groups of microorganisms were the genera *Bacillus* and *Serratia*. A significant effect on the composition of the cultured microbiota was caused by lycopene (in all studied concentrations), leading to a significant increase in the relative abundance of *Firmicutes* in the anterior gut. Studies of the histological structure of the anterior and posterior guts have shown the relationship between the barrier and secretory functions of the gut and the composition of the microbiota while using butyric acid (1 and 2 g kg^−1^) and trace element chelated compounds (2 mg kg^−1^). This culture-dependent method of studying the microbiome makes it possible to assess changes in some representatives of the main groups of microorganisms (*Firmicutes* and *Proteobacteria*). Despite the incompleteness of the data obtained by the culture-dependent method, its application makes it possible to assess the bioactive properties of feed and feed additives and their impact on the microbiota involved in digestive processes.

## 1. Introduction

Increasing the productivity of industrial aquaculture is possible through the use of special additives in the production of feeds, which have a whole range of useful functional properties [1,2]. Among the additives that are currently used are vitamin and trace element premixes, probiotics, and various biologically active substances (antioxidants, growth stimulants, etc.) [3,4,5,6,7]. The performance of supplementation is assessed by a number of parameters, typically gastrointestinal (GI) response, blood biochemical and immune parameters, and growth metrics [8,9,10,11,12].

The GI microbiota plays an important role in digestion, immunity maintenance, and gut barrier function [13,14,15]. The state of the microbiota can also serve as an important indicator in the study of feed additives [16]. Composition of the gut microbiota can directly affect the overall health of aquaculture species as shifts in microbiome composition can lead to various diseases [15,17]. Current data show that the fish microbiome mainly includes phyla such as *Proteobacteria*, *Firmicutes*, *Bacteroidetes*, *Fusobacteria*, and *Actinobacteria* [18,19,20]. The abundance of these microorganisms as well as the overall taxonomic diversity depends on a number of exogenous factors [21,22], among which feeding is one of the key factors [23].

Among the feed additives, pro/prebiotic supplements have the greatest impact on gut microbiota [24,25]. To date, the evaluation of the effects of organic and inorganic feed additives on gut microbiota has been actively studied in both aquaculture species and laboratory fish [26,27,28,29]. Some feed additives are rapidly absorbed in the GI tract without significantly affecting the composition of the commensal microbiota. Nevertheless, they can inhibit or stimulate the development of certain groups of microorganisms, which has been demonstrated in several studies [21,30,31,32].

In this study, organomineral compounds (trace elements) in chelated form (CH), butyric acid (BA), and lycopene (LYC) were used as promising feed additives. These feed components are actively used in other areas of agriculture and have proven functional properties [33,34]. Each of these feed additives is characterized by different mechanisms of adsorption and incorporation into the metabolism. This suggests their unique influence on the composition of the intestinal microbiota. Organomineral chelated micronutrient compounds are a source of micronutrients and have greater bioavailability compared to inorganic salts [35,36]. Butyric acid, which is a postbiotic drug, has positive effects on gut microbiota and intestinal health [37]. In addition, butyric acid is constantly present in the gut as a product of the metabolism of commensal bacteria, acting as an important source of energy for the intestinal epithelium [38]. Lycopene is a promising antioxidant with a wide range of biological activities (anti-inflammatory, detoxification, prevention of cardiovascular disease) [39,40]. Lycopene is widely used both in the food industry [33] and in aquaculture [41]. Carotenoids are widely used in aquaculture because they affect the health, coloration, and quality of the meat [12].

Research on the effects of different feeds and feed components is convenient to carry out on model objects. One of the most common test objects is *Danio rerio* (*D. rerio*), which is widely used for toxicological, biomedical, and genetic studies [42,43,44]. *D. rerio* can be used as an experimental model to develop new feed recipes for commercial aquaculture facilities [45]. The studies performed on *D. rerio* have shown that the most represented groups of intestinal-associated microorganisms are *Proteobacteria*, *Firmicutes*, and *Fusobacteria* [43,46]. In addition, many authors note the existence of variations in the composition of the microbiome in distinct parts of the intestine due to physiological differences in the digestion and absorption of nutrients [47]. Some authors believe that the main absorption of nutrients in *D. rerio* occurs mainly in the anterior gut, where a large number of digestive enzymes are present [43,48].

A lower diversity of gut-associated microbiota has been described for laboratory-cultured fish compared to wild specimens [46]. This is because both exogenous (host taxonomy) and endogenous factors are involved in the formation of the gut microbiome; in particular, endogenous factors for laboratory fish can include the use of artificial feed [49,50], the control of the presence of pathogenic bacteria, and the poor taxonomic composition of aquatic microorganisms [51,52]. Various culture-dependent and culture-independent techniques are used to study the gut microbiome. The use of culture-dependent techniques allows only part of the gut bacterial community to be identified, not always reflecting the real taxonomic diversity of the microbiome due to a lack of necessary medium selectivity and the unculturable nature of some microorganisms, bacterial competition for nutrient media, suppression of some bacteria by others, and the inability to culture dead or damaged cells [53,54,55]. Although molecular genetic methods provide a more complete taxonomic picture, it should be taken into account that they cannot always distinguish closely related groups of microorganisms [56], are limited in the study of rare species, and are unable to detect variations at the species level [57]. Based on this, it can be suggested that culture-dependent techniques can be used to establish the main shifts in the relative abundance of microorganisms when investigating the effects of the feed components and additives on the commensal microbiota of aquaculture fish species. The absence of significant changes in the microbiome when using feed components indicates the safety of the developed feed formulations for aquaculture in the case of production feeds. In contrast, if the feed is developed for the prevention and correction of the gut microbial community (probiotic preparations, treatment of diseases), positive shifts in the microbiome should be confirmed.

The main part of the microbiome involved in the process of digestion, in contrast to the transition microbiota, is located directly on the intestinal mucosa and, thus, participates in the processes of the adsorption of nutrients. Changes in intestinal tissue morphology may be related to the composition of the commensal microbiota. The combined study of GI histology and the taxonomic composition of the microbiota allows for a more accurate assessment of the effect of feed additives [43].

Thus, the present work intended to study the effect of the use of three feed additives with different functional properties on the culturable microbiota of the anterior and posterior sections of the intestine of *D. rerio*. The effect of these substances on the morphological structure of the two sections of the intestine was also studied. In addition, this work considered the possibility of using culture-dependent methods to study changes in the intestinal microbiota of fish in the evaluation of feed and feed additives.

## 2. Materials and Methods

### 2.1. Animals and Experimental Conditions

All studies were performed in accordance with the guidelines of the Local Ethics Commission of the Institutional Review Board of the Moscow State University of Technology and Management (approval number 4, 1 March 2022).

Adult *D. rerio* at three months old, 2.03 ± 0.17 cm in size, and 0.26 ± 0.03 g in weight, which were kept in 50 L aquariums at 24 ± 1 °C and pH 7.2 ± 0.2 under natural light conditions (12 h); 25 animals in triplicate (*n* = 3), according to standard maintenance protocols [58], were used as test objects. The duration of the experiment was 60 days. The water in the experimental group tanks was replaced partially (1/3) every three days, and fully every seven days. Feeding was carried out three times a day.

### 2.2. Feed Preparation

The experiment included three experimental (BA, CH, LYC) and one control group (Con). Coppens vital 0.5–0.8 mm commercial pelleted feed (Coppens, Helmond, Netherlands) was used as the basal feed mixture. Butyric acid (BA) in concentrations (g kg^−1^) of 0.5, 1.0, and 2.0 [59,60] and organomineral chelated trace element compounds (CH) (Jupiter LLC, Tver, Russia) (composition of the mother liquor g L^−1^: Fe—10; Mn—15; Zn—35; Se—0.3; I—1.1; Cu—3) in concentrations (g kg^−1^) of 0.5, 1.0, and 2.0 [61,62,63] were incorporated into the base feed by microencapsulation with sodium alginate. For this purpose, sodium alginate was dissolved in distilled water (0.5 mg 100 mL^−1^), and then the test additives in the described concentrations were added to the solution. The prepared solution was evenly applied to the feed by spraying. The feed was then dried to its original humidity at 40 °C. The control feed was prepared according to the scheme described above (adding sodium alginate) without adding feed additives.

Lycopene extract (LYC), as a fat-soluble compound, was dissolved in 20 mL of corn oil. The following concentrations of lycopene (mg kg^−1^) were used in the experiment: 25, 50, and 75 [41,64]. To obtain a lycopene solution in corn oil, the following concentrations of the extract were dissolved 2.5; 5 and 7.5 mg (for 100 g of feed). The resulting solution was evenly distributed throughout the feed used in the experiment.

The prepared feeds were stored at 4 °C in a refrigerated chamber. The experimental feed was prepared anew on the 30th day in order to preserve the functional properties of the feed additives. Since the addition of corn oil changes the lipid composition of the resulting feed mixture, the same amount of oil (20 mL) was added to the feed of all of the other experimental groups.

### 2.3. Sampling, Bacterial Isolation, and Identification

At the end of the experiment (on the 60th day), three fish were randomly selected from each experimental group and placed in separate containers. For three days, the fish did not receive feed, which contributed to the elimination of feed residues and transit bacteria from the intestines. This procedure was necessary to determine their own commensal microbiota and to minimize the bacterial background produced by the feed.

Bacterial sampling was performed from two sections of the intestine, anterior (including middle) and posterior [47,48]. Microbiological sampling was performed by the direct method (hot sealing) on prepared Petri dishes. Before this, the fish were sedated in MS-222 solution (10 mg L^−1^), then the external coverslips were disinfected under sterile conditions and dissected.

A nutrient medium satisfying the purposes of analysis of cultured microbiota of the intestines of *D. rerio* was used, Endo-Agar (Endo-GRM). This nutrient medium was chosen because of its selective property (suppression of Gram-negative microorganisms). The results of the inoculations from this type of media aimed at determining the presence of Enterobacteriaceae group microorganisms and the selection of coliform group bacteria. Brian heart infusion (BHI) agar was also used in the study and has selective and nutritional properties that allow for a wide range of microbiota to be examined.

After microbiological inoculation, the Petri dishes were placed in a Binder BF 56 thermostat (Binder, Tuttlingen, Germany) for seven days at a constant temperature of 24 °C, corresponding to the water and fish body temperature, for further incubation. Bacterial growth was monitored daily (see the Section 2.4).

After the growth of the first colonies, pure cultures were isolated for the further determination of taxonomic identification. For this purpose, characteristic daily colonies were selected and transferred to Petri dishes with the same type of nutrient media. One day after transplantation of the pure cultures, primary systematic identity tests (Gram stain, cytochrome oxidase test, catalase reaction test, urease) and microscopy (bacterial cell size, motility, morphology, character of grouping) were performed. A coagulase activity test was performed for Gram-positive cocci. The analysis of Gram-negative bacteria included indol production, the Voges–Proskauer reaction, methyl red test, and possibility of using citrate as a carbon source. For most of the isolated bacteria, the possibility of the degradation of glucose, lactose, and maltose was also evaluated (Table S1).

A visual examination of the bacterial colonies on nutrient media was also performed to look for characteristic features (morphology of colonies, reaction of indicator components of nutrient media on bacterial cultures, and consideration of the differentiation property of media reaction). All of the defined morphological and biochemical characteristics of the bacterial colonies were compared with Bergey’s Manual of Determinative Bacteriology, Ninth Edition (2000).

### 2.4. Semi-Quantitative Assessment of Bacterial Abundance

The number of bacteria in each systematic group was estimated semi-quantitatively according to the method described by Noakes [65] and Cantas [23]. The number of colonies on the dishes was counted as follows: 0 (none); <10 (1, very few); 10–50 (2, few); 50–100 (3, moderate); 100–200 (4, rich); >200 (5, very rich).

### 2.5. Histology

For histological sections, three individuals without visible lesions were selected from each experimental group. Fish were immortalized in MS-222 solution (10 mg L^−1^), after which they were fixed in Davidson’s solution for 24 h. Then, tissue samples were dehydrated in a series of graded alcohols and embedded in paraffin. To obtain sections of the anterior/middle and posterior intestines of each fish, serial slices (4 μm) in the frontal plane were made and stained with hematoxylin and eosin (H&E). Histological sections were prepared and stained according to Suvarna et al. [66]. In addition, morphometric measurements of the intestines of three specimens of *D. rerio* (50 measurements per section) were performed according to the following parameters: height of the adsorbing epithelium, width of lamina propria, goblet cells area, number of goblet cells per 100 µm of epithelium, and thickness of the muscularis.

Histological preparations were examined under an Olympus BX53 light microscope (Olympus Corporation, Tokyo, Japan) with CarlZeiss ERc 5 s (Zeiss, Oberkochen, Germany) and ToupCam 16.0 MP (ToupTek Photonics, Hangzhou, China) ocular attachments using ZEN lite software (Zeiss, Oberkochen, Germany) and ToupCam view 16.0 (ToupTek Photonics, Hangzhou, China).

### 2.6. Statistical Analysis

Data comparing the relative abundance of microorganisms are presented as the mean ± SD; statistical significance was determined using the Mann-Whitney test (*p* value < 0.05 was taken as statistically significant). The correlation between the relative abundance of microorganisms and concentrations of feed additives was determined using Pearson’s correlation, and Student’s distribution was used to compare the slopes and intercepts. Statistical data were processed using GraphPad Prism version 8.0 software (GraphPad, San Diego, CA, USA).

## 3. Results

### 3.1. Cultured Intestinal Microbiota

An investigation of the culturable intestinal microbiota of *D. rerio* when using the studied feed additives revealed the presence of changes in the representation of systematic groups of microorganisms and their relative occurrence.

**Figure 1:**
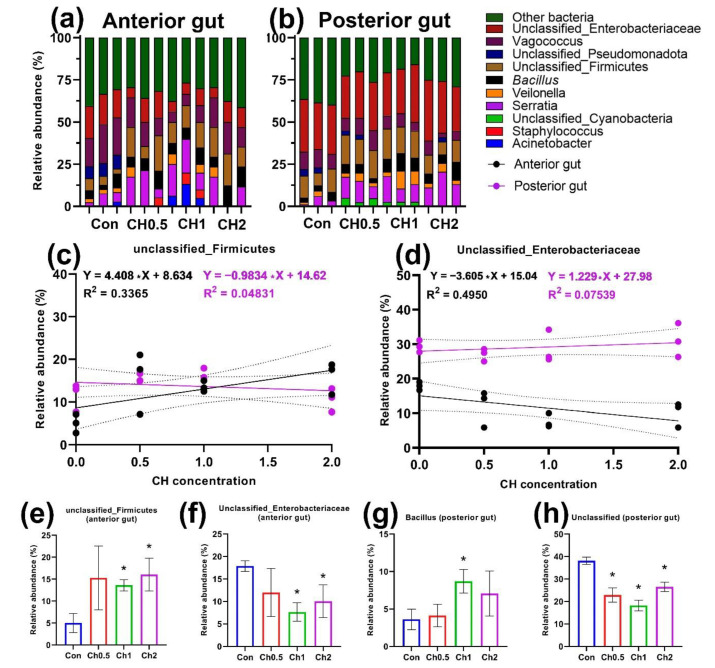
Cultivated microbiota of the anterior and posterior sections of the intestine of the experimental groups of *D. rerio* receiving chelated compounds of trace elements (*n* = 3). Unclassified bacterial groups are labeled within defined taxonomic groups: (**a**,**b**) relative abundance of microbiota by bacterial genus in the anterior and posterior gut sections, respectively; (**c**,**d**) linear regression of the relative abundance of bacteria from unclassified_*Firmicutes* and unclassified_*Enterobacteriaceae* groups and CH concentration in the anterior (black dots) and posterior (purple dots) sections; (**e**–**h**) relative abundance of different bacteria groups in the control and experimental groups. The value (*—*p* < 0.05) from the Mann-Whitney test.

Differences in the microbiota of specific sections of the intestine were noted (raw data are presented in the Table S3). In the anterior intestine of the control group, there were eight groups of microorganisms, of which five were identified for genus affiliation (*Acinetobacter*, *Vagococcus*, *Serratia*, *Bacillus*, *Veilonella*) and three were unclassified and assigned to different taxonomic groups (unclassified_*Enterobacteriaceae*, unclassified_*Firmicutes*, unclassified_*Proteobacteria*) (Figure 1a and Figure S1). In the hindgut, the taxonomic composition of the cultured microbiota was distinguished by the absence of the genus *Acinetobacter* (Figure 1b). At the type level, *Firmicutes* (33.46; 25.03%) and *Proteobacteria* (31.74; 35.34%) dominated the cultured microbiota of the anterior and posterior regions. Undetermined bacteria (other bacteria) were represented in 34.78% and 34.16% relative to other microorganisms for the anterior and posterior intestines, respectively.

By comparing the experimental groups with the control individuals, the following differences were revealed. Micronutrient chelated compounds in the studied concentrations had an effect on the cultivated microbiota of both the anterior and posterior sections of the intestine. The bacteria of the genera *Serratia* and *Veilonella* practically did not change compared to the control, both in the anterior and posterior sections of the intestine. Relative abundance of the genus *Vagococcus* did not change significantly in all of the studied concentrations.

In the anterior intestine, the effect of micronutrient chelated compounds significantly led to a change in the relative representation of unclassified *Firmicutes* and *Enterobacteriaceae*. In the CH1 and CH2 groups, there was a significant (*p* < 0.05) increase in the frequency of unclassified *Firmicutes* and a decrease (*p* < 0.05) in the representation of unclassified *Enterobacteriaceae* (Figure 1e,f). The relative abundance of *Firmicutes* was found to increase with increasing CH concentrations (R^2^ = 0.336) (Figure 1c). At the same time, the abundance of this group did not change significantly in the posterior intestine. The slopes of the regression lines for the anterior and posterior parts of the intestine for unclassified__*Firmicutes* and unclassified__*Enterobacteriaceae* bacteria were significantly different. (*p* < 0.05).

In the posterior intestine, there was a significant increase (*p* < 0.05) in the relative abundance of the genus *Bacillus* in the CH1 group (Figure 1b). The occurrence of undetermined bacteria («Other bacteria» in the Figure 1a,b) significantly decreased (*p* < 0.05) in all of the studied groups (Figure 1d).

**Figure 2:**
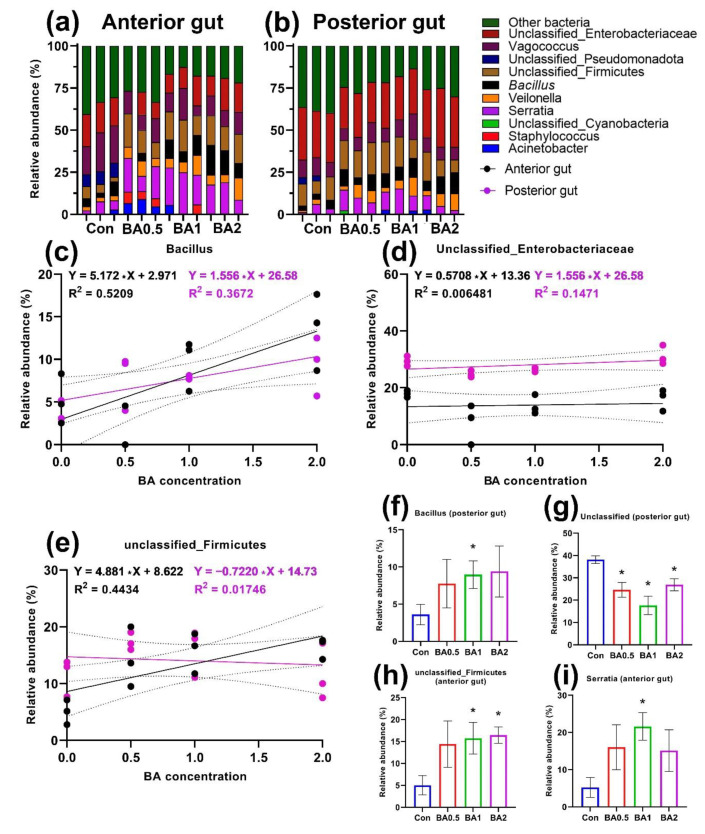
Cultivated microbiota of the anterior and posterior sections of the intestine of the experimental groups of *D. rerio* receiving butyric acid (*n* = 3). Unclassified bacterial groups are labeled within defined taxonomic groups. Unclassified bacterial groups are labeled within defined taxonomic groups: (**a**,**b**) relative abundance of microbiota by bacterial genus in the anterior and posterior gut sections, respectively; (**c**–**e**) linear regression of the relative abundance of *Bacillus*, unclassified_*Enterobacteriaceae* and unclassified_Firmicutes groups and BA concentration in the anterior (black points) and posterior (purple points) sections; (**f**–**i**) relative abundance of different bacteria groups in the control and experimental groups. The *p*-value (*—*p* < 0.05) from the Mann-Whitney test.

Butyric acid had an effect on the cultured microbiota of both sections of the intestine, mainly at concentrations of 1 and 2 mg kg^−1^. The anterior part of the intestine was dominated by undetermined bacteria as well as microorganisms of the groups unclassified_*Firmicutes*, *Vagococcus*, and *Serratia* (Figure 2a). At the same time, *Serratia* and *Firmicutes* showed a significant increase in representation compared with the control group (Figure 2h,i). Moreover, the relative occurrence of *Serratia* correlated (R^2^ = 0.4434) with the concentration of BA in the foregut (Figure 2e). Relative abundance of unclassified *Enterobacteriaceae* in both the anterior and posterior gut sections showed no correlation (R^2^ = 0.006; 0.147) with the concentration of butyric acid (Figure 2d). Genus *Bacillus* in both gut sections showed a positive correlation with the butyric acid concentration (R^2^ = 0.52; 0.367) (Figure 2c). However, a significant difference was found (*p* < 0.05) only in the hindgut in the BA2 group. The slope of the regression lines for the relative abundance of bacteria in the anterior and posterior parts of the intestine was different (*p* < 0.05) only for unclassified_*Firmicutes*. The relative abundance of the undetermined bacteria was significantly lower in the experimental groups compared to the control (*p* < 0.05) (Figure 2g).

**Figure 3:**
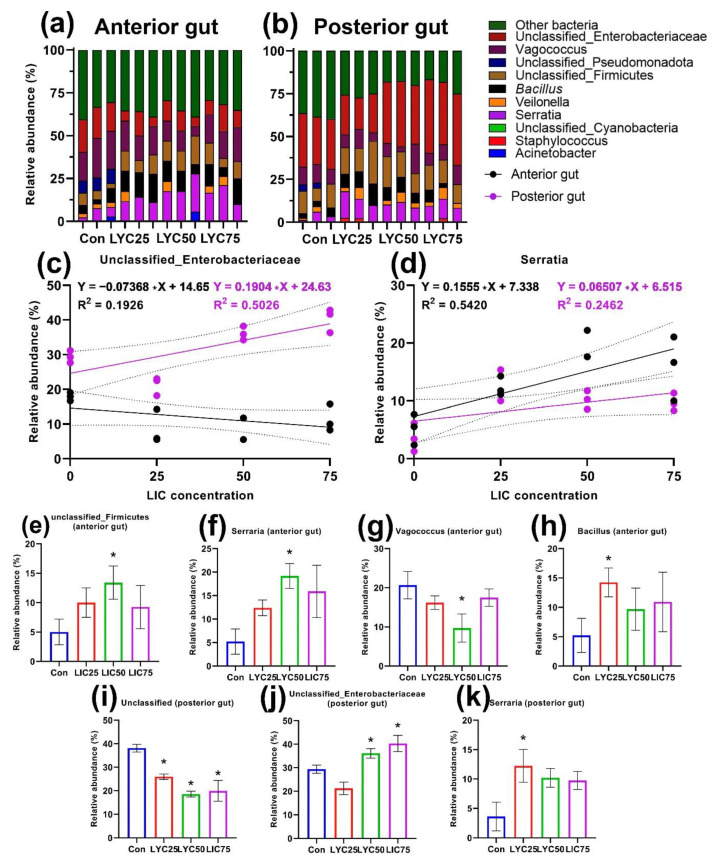
Cultivated microbiota of the anterior and posterior sections of the intestine of experimental groups of *D. rerio* receiving lycopene (*n* = 3). Unclassified bacterial groups are labeled within defined taxonomic groups: (**a**,**b**) relative abundance of microbiota by bacterial genera in the anterior and posterior intestinal sections, respectively; (**c**,**d**) linear regression of the relative abundance of unclassified_*Enterobacteriaceae* and *Serratia* in LYC concentration in the anterior (black points) and posterior (purple points) intestinal sections; (**e**–**k**) relative abundance of different bacterial groups in the control and experimental groups. Value (*—*p* < 0.05) from the Mann-Whitney test.

The administration of lycopene in the feed for *D. rerio* caused significant changes in the composition of the cultured gut microbiome. In the anterior gut, a significant increase in the representation (*p* < 0.05) of bacteria of the genera *Bacillus*, *Serratia*, *Vagococcus*, and unclassified *Firmicutes* was found in the LYC25 and LYC50 groups, respectively (Figure 3e–h). At the same time, the genus *Serratia* was also significantly different from the control (*p* < 0.05) in the posterior region, showing a positive dose-dependent effect only in the anterior region (R^2^ = 0.542) (Figure 3d). The slope of the regression lines of the anterior and posterior regions of the genus *Serratia* was not significantly different. Supplementation with lycopene in the feed caused minimal changes in the posterior intestine microbiota. A positive dose-dependent effect relationship could also be observed for the unclassified_*Enterobacteriaceae* in the same gut region (R^2^ = 0.5026) (Figure 3c). The slope of the regression lines was also significantly different (*p* < 0.01) between the anterior and posterior parts of the intestine. The group of undetermined bacteria decreased (*p* < 0.05) its abundance in all groups with the addition of LYC in the posterior intestine (Figure 3i).

### 3.2. Intestinal Histology

**Figure 4:**
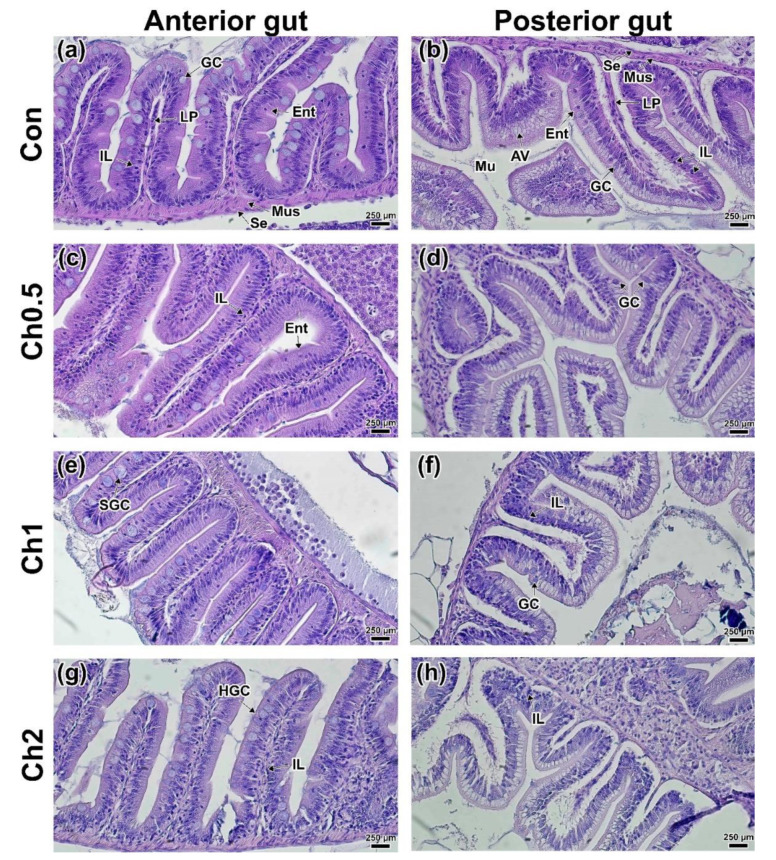
Histology of the anterior (**a**,**c**,**e**,**g**) and posterior (**b**,**d**,**f**,**h**) intestines of *D. rerio* of the experimental groups receiving chelated compounds of microelements and control: (**a**,**b**) control group; (**c**,**d**) CH0.5 group; (**e**,**f**) CH1 group; (**g**,**h**) CH2 group. Mus—muscularis; SE—serous layer; Lu—lumen; Mu—mucosa; LP—Lamina propria; GC—goblet cells; EN—epitheliocyte nucleus/columnar pseudostratified epithelium; AV—supranuclear absorptive vacuole; IL—intraepithelial leucocytes; EC—esonophilic/immune cells; Ent—basophilic enteroendocrine; HGC—hypertrophy-like goblet cell; SGC—swelling goblet cell. H&E staining, scale bar 250 µm.

The study of the intestine histology of experimental individuals of *D. rerio* showed the absence of pronounced morphological changes in the tissue structure. Intestinal sections of *D. rerio* from all groups showed clearly defined intestinal villi including absorbing epitheliocytes, goblet cells, basal membrane, and lamina propria (LP). The outer layer of the intestine included serous and muscular layers (Figure 4). The intestine of *D. rerio* consists of three sections without a distinct stomach. The anterior intestine differed in the greater height of the villi and the number of goblet cells as well as in the thickness of the muscular and serous layers (Figure 4a). The posterior intestine has a smaller lumen, fewer goblet cells, and lower villous height (Figure 4b). In addition, epitheliocytes of this part of the intestine have weak basophilic staining due to the presence of absorbing vacuoles in the cytoplasm [67]. A small number of enteroendocrine cells was also recorded in the posterior part of the intestine.

The minimum concentration of CH (0.5 mg kg^−1^) had no significant effect on the studied intestinal parts. There was a lower occurrence of enteroendocrine cells in some parts of the anterior intestine (Figure 4c). No morphological changes were found in the posterior region (Figure 4d). In the CH1 and CH2 groups, similar histological differences from the controls were observed. Thus, an increase in the size and number of goblet cells was observed in most of the anterior mucosa (Figure 4e,g). In the posterior intestine, a large number of intraepithelial lymphocytes was observed in both the interstitium (LP) and in the basal part of enterocytes (Figure 4f,h). The findings were confirmed by morphometric measurements of the lamina propria, the width of which was significantly increased (*p* < 0.05) from the control in the CH2 group (Table S2). No signs of intestinal epithelium degradation were observed in any of the groups fed CH.

**Figure 5:**
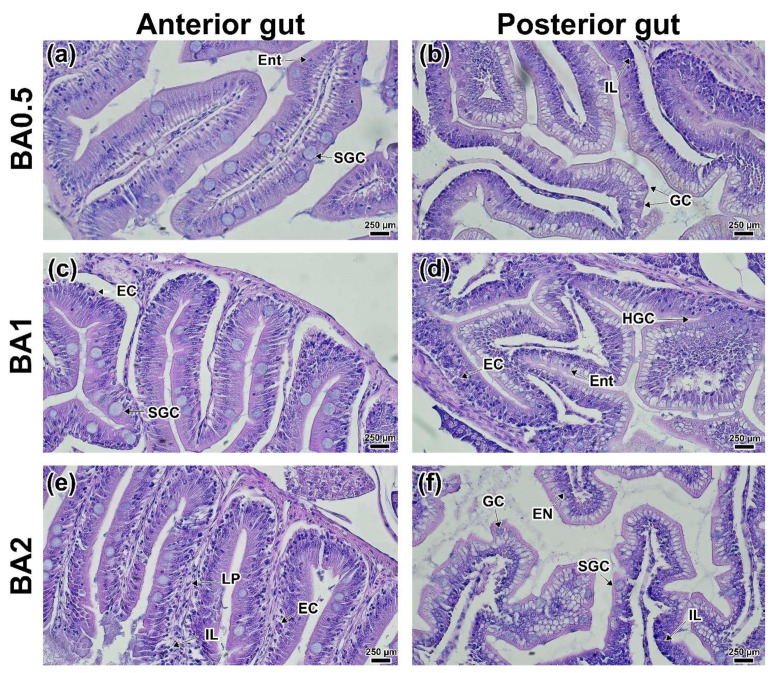
Histology of anterior (**a**,**c**,**e**) and posterior (**b**,**d**,**f**) intestines of the *D. rerio* experimental groups receiving butyric acid: (**a**,**b**) BA0.5 group; (**c**,**d**) BA1 group; (**e**,**f**) BA2 group. LP—Lamina propria; GC—goblet cells; EN—epitheliocyte nucleus/columnar pseudostratified epithelium; IL—intraepithelial leucocytes; EC—esonophilic/immune cells; Ent—basophilic enteroendocrine; SGC—swelling goblet cell. H&E staining, scale bar 250 µm.

In the anterior intestine of the BA0.5 group, there was a large number of enlarged goblet cells occurring throughout the mucosa (Figure 5a). A significant difference (*p* < 0.05) in the goblet cell area from the control was observed (Table S2). At the same time, the total number of enteroendocrine cells was lower than in the control. An increase in the number of goblet cells as well as the migration of intraepithelial leukocytes was observed in some areas of the posterior intestinal mucosa (Figure 5b). Similar changes were observed in the BA1 group. In addition, eosinophilic cells, predominantly located in the lower part of the LP villi, were also found in this group (Figure 5c). Eosinophilic cells were also found in the posterior intestine (Figure 5d). Morphological differences from the controls were more severe at the maximum concentration of butyric acid (BA2). In the anterior intestine, intraepithelial leukocytes and eosinophilic cells were observed throughout the mucosa, which was also seen in as a significant increase in the LP thickness (*p* < 0.05; Table S2). Additionally, in the posterior intestine, there were signs of hypertrophy of the enterocyte nuclei (Figure 5e,f).

**Figure 6:**
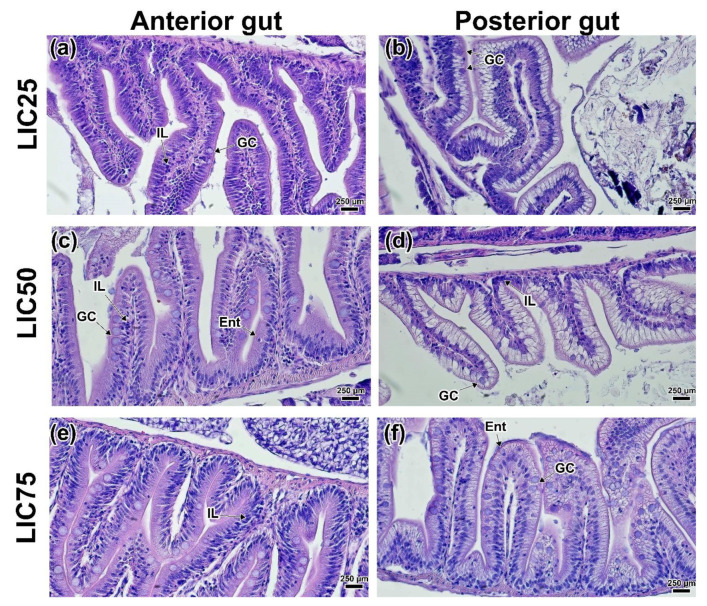
Histology of the anterior (**a**,**c**,**e**) and posterior (**b**,**d**,**f**) intestines of the *D. rerio* experimental groups receiving lycopene: (**a**,**b**) LYC25 group; (**c**,**d**) LYC50 group; (**e**,**f**) LYC75 group. LP—Lamina propria; GC—goblet cells; EN—epitheliocyte nucleus/columnar pseudostratified epithelium; IL—intraepithelial leucocytes; Ent—basophilic enteroendocrine. H&E staining, scale bar 250 µm.

Supplementation of lycopene at all of the concentrations studied did not result in significant morphological changes in the intestine of *D. rerio*. Thus, in the LYC25 and LYC50 groups, there was an increase in the number of intraepithelial leukocytes in the mucosa (Figure 6a,c). A lower number of enteroendocrine cells was observed only at the minimum concentration of lycopene (25 mg kg^−1^). In addition, a significant decrease in the goblet cell area (*p* < 0.05) in the LYC75 group should be noted (Table S2). The posterior intestine did not differ from the control except for clusters of intraepithelial cells in some areas of the mucosa and interstitium (Figure 6b,d). The LYC75 group had minimal differences from the controls. An increase in the number of intestinal endocrine cells (mucoid, enteroendocrine) could be seen in the hindgut (Figure 6f).

## 4. Discussion

Examination of the effect of feed additives on the composition of the culturable gut microbiota of *D. rerio* showed significant changes in the composition of the bacterial community. All of the supplements studied showed activity against the gut microbiome of *D. rerio*. Differences were also detected between the anterior and posterior guts. It should be noted that none of the studied additives showed pronounced bacteriostatic properties. The presence of the effect of feed additives, even in minimal concentrations, is associated with a deep interrelationship between groups of microorganisms, where even a slight impact leads to a structural change in the microbiocenosis.

Previously, it has been shown that changes in the feed composition and feeding regimen are a modulating factor for the microbiome due to its ability to adapt [49]. In this study, all changes occurring in the microbiome were associated with changes in the ratio of individual groups of microorganisms, especially with the use of butyric acid, which proves its postbiotic properties. Microorganisms of the genera *Bacillus* and *Serratia* as well as unclassified_*Enterobacteriaceae* showed the greatest variability in terms of abundance according to the results of our studies. Changes in the composition of these groups under different types of exposure have been noted in the works of other authors [46,68,69]. Although changes in microbial composition can occur in a short time, a long experiment (60 days) allowed us to establish a stable picture of the formed microbiome.

Micronutrients are an important component of feed as they are necessary for the proper growth and development of fish, the maintenance of immunity, and meat quality [70]. Organic compounds of trace elements have been shown to exhibit greater bioavailability compared to their inorganic analogs, resulting in the intensification of growth-weight and biochemical parameters [7]. Recent data show that the gut microbiota is involved in the metabolism of the most bioactive trace elements [71]. In the available works on the effect of trace elements (organic and inorganic) on the gut microbiome of fish and other animals, the effect on the quantitative and qualitative indicators of the microbiota has also been found [72,73,74]. However, these studies have mainly examined the effect of individual trace elements, while the combined effect of several trace elements has been investigated only partially. Most likely, this is due to the difficulty of interpreting the data.

Our work showed that groups of microorganisms such as *Serratia*, *Veilonella*, and *Vagococcus* were not sensitive to the presence of CH in the feed. In our opinion, iron and zinc play the greatest physiological role, although other trace elements are also necessary for the development of some bacterial groups. High concentrations of chelates (1 and 2 mg kg^−1^) inhibited the development of coliform bacteria in the anterior segment, leading to increased numbers of *Firmicutes*. There was also an increase in the relative abundance of the genera *Serratia* and *Bacillus* in the hindgut. A number of studies have shown that the ratio of Gram-positive (e.g., *Firmicutes*, *Actinobacteria*) to Gram-negative (*Proteobacteria*, *Bacteroidetes*) bacteria correlate with organism health [75,76]. When *D. rerio* was exposed to aluminum, changes in the ratio of these groups toward an increase in the number of Gram-negative bacteria (*Fusobacteriaceae*) and a decrease in *Firmicutes* (predominantly *Bacillus* genus) were recorded [77]. Similar shifts in the composition of the microbiota have been observed with exposure to other heavy metals [78] and toxic components [22,79,80]. In this case, we can talk about the opposite effect of CH on the gut microbiota in the anterior region.

According to several authors, organic iron is intensively assimilated by the host, and only a small part of it is consumed by the iron-dependent bacterial groups [81]. Zinc deficiency, in turn, can lead to a decrease in *Firmicutes* and an increase in *Proteobacteria* populations [71]. As mentioned previously, low concentrations are absorbed almost immediately in the fish’s GI tracts, showing weak effects on the intestinal microbiota, especially in the posterior intestine. Free chelating agents can bind to other metal ions, creating a local imbalance of trace element composition in the intestinal mucosa of fish. A high availability of chelated compounds of trace elements can be assumed to promote their assimilation, not only by mucosal epithelicytes but also by intestinal bacteria, leading to the stimulation of their growth at high concentrations of organomineral premix. Thus, shifts in the relative abundance of gut microorganisms caused by the use of chelated compounds do not lead to an increase in coliform bacteria, which probably has a positive effect on gut health.

The obtained data were confirmed by histological studies. Low concentrations of chelated compounds did not cause changes in the morphological parameters of the intestinal villi of the anterior and posterior regions. At concentrations of 1 and 2 mg kg^−1^, there was an increase in the area of goblet cells in the anterior intestine and the presence of intraepithelial leukocytes in the posterior intestine. In our previous studies of the use of chelated compounds in low concentrations, similar histomorphological changes in the intestine (stimulation of immune cell migration into the mucosa, hypertrophy of goblet cells) were detected [61,62]. Perhaps such morphological changes in the intestines may be associated with lower toxicity and greater bioavailability of the chelated compounds. In general, we can conclude that CH in the studied concentrations does not lead to histological alteration in either the anterior or posterior parts of the intestine of *D. rerio*.

Butyric acid as well as a number of other short-chain fatty acids are metabolites of Firmicutes as well as several other groups of bacteria (*Ruminococcaceae*, *Eubacterium*, *Clostridia*) [38]. The application of butyric acid in feed resulted in changes in the quantitative indices of the cultured microbial community of the anterior gut. An increase in the relative abundance of the genus *Bacillus* and the unclassified_*Firmicutes* group was directly correlated with BA concentration. At the same time, no similar correlation was observed in the posterior gut. This is most likely due to the fact that the greatest short-chain fatty acid adsorption activity occurs in the anterior intestine, which influences the growth of these groups of microorganisms [82]. In addition, it is worth noting that the genus *Bacillus* is a common group of bacteria in the intestine of *D. rerio* [43]. Most of the probiotics studied in aquaculture belong to the *Firmicutes* phylum, namely *Lactobacrea* and *Bacillus* spp. [83,84,85,86]. The probiotic effect of these groups of bacteria is manifested in a synthesis of biologically active compounds that improves the barrier function of the intestine and stimulates the immune response as well as the displacement of opportunistic pathogens [87,88]. On histological sections of the anterior region, there was a stimulation of the immune response expressed in an increase in the number of immune cells in the interstitium (intraepithelial leukocytes, eosinophilic cells), which has also been shown in other works [89].

The intake of additional BA with feed may have contributed to the inhibition of individual bacterial groups. One of the ways short-chain fatty acids are adsorbed by the host is through dissociation in exchange for HCO_3-_, which decreases the pH in the intestinal lumen [82,90]. In studies on other animals, it has been shown that low pH values lead to changes in the composition of the microbiota, preventing the growth of pH-dependent bacteria (e.g., *Enterobacteriaceae*) [91,92]. This is in agreement with our results, where the relative occurrence of unclassified_*Enterobacteriaceae* did not change. In addition to the stimulation of the microbiota and the immune response of the gut, BA led to an increase in the area of goblet cells in the anterior region and an increase in their number in the posterior region. This may be related to both the direct response of the gut to changes in the qualitative composition of the microbiome and to the direct effect of butyric acid on enteroendocrine cell secretion [38]. Thus, it has been shown that the inclusion of various pro/prebiotic supplements in feed composition leads to changes in gut secretory activity (goblet and enteroendocrine cells) as well as the activation of intraepithelial leukocytes [93]. The increased production of mucin can be interpreted as a positive effect since the layer of this glycoprotein separates the apical part of mucosal epitheliocytes and the commensal microbiota [94]. Thus, we can conclude that butyric acid leads to a significant increase in Firmicutes representatives in the anterior region, which also leads to an increase in immune cells in the intestine and stimulates mucin secretion.

The application of lycopene in the feed had a significant effect on the cultured gut microbiome of *D. rerio*. Similar to the other studied feed additives, lycopene led to an increase in the relative abundance of *Firmicutes* in the anterior region, with unclassified_*Firmicutes* having a positive correlation with LYC concentration. In the posterior region, there was no significant effect on the *Firmicutes* phylum. Since carotenoids have different bioavailability, their absorption can probably occur along the entire length of the fish GI tract [95]. The interaction between carotenoids and the gut microbiota is poorly understood [96]. It is possible that the gut microbiota is involved in carotenoid metabolism, as Nguyen et al. [12] demonstrated a relationship between trout meat color and the microbiological profile of different gut segments. In our work, a significant rearrangement of the microbial community was recorded only in the anterior region. The *Proteobacteria* phylum, in turn, demonstrated a positive correlation between the relative occurrence of LYC concentration in the anterior (*Serratia*) and posterior (unclassified_*Enterobacteriaceae*) sections, respectively. *Proteobacteria*, although they are always represented in the GI tracts of both warm-blooded and cold-blooded animals, play an ambiguous role from typical saprophytes involved in digestive processes [97] to conditionally pathogenic strains involved in the development of GI diseases [98]. Increased representation of *Enterobacteriaceae*, in our opinion, does not affect the health of fish, as they are a normal component of the hindgut microbiota [12]. In addition, these groups of bacteria are important for maintaining gut homeostasis and regulating physiological processes, particularly digestion [68,99].

Our findings do not strictly coincide with the opinions of other authors [100], who have studied the effect of beta-carotene on the microbiota of *Pyropia columbina*, although this work also recorded changes in the microbial community at 60 days of feeding. The increase in the relative occurrence of *Firmicutes* and *Proteobacteria* may also be related to the ability of lycopene to inhibit the actions of specific antibacterial compounds [101].

Adsorption of fat-soluble carotenoids occurs on the apical part of the villi by trapping mixed micelles or from carrier proteins using membrane transporters (SR-BI, CD36, and NPC1L1) [94]. Since microbiota have previously been shown to be involved in lipid metabolism [69], we can speculate that microorganisms may be involved in carotenoid adsorption (combining carotenoids with lipids and their metabolic products into micelles) [102]. At this time, there are almost no studies on the effect of lycopene on the fish microbiota. The available studies on higher vertebrates and humans demonstrate the promise of its use. In addition, we can assume that the antioxidant activity of lycopene affects both the barrier function of the gut [102] and the development of certain groups of microorganisms (*Firmicutes*, e.g., *Clostridiales*, *Lactobacillus*, and *Actinobacteria*, e.g., *Bifidobacterium*) [80,103].

Lycopene had the least effect on the histomorphological parameters of the *D. rerio* intestines among all of the studied feed additives. An increase in the number of intraepithelial leukocytes and a decrease in the occurrence of enteroendocrine cells observed in some areas of both studied sections are most likely related to the individual characteristics of the fish. The absence of visible histomorphological changes suggests that higher concentrations must be used to obtain a more significant effect [104]. The obtained data allowed us to conclude that the cultured microorganisms were more sensitive to the action of lycopene than the histological structures of the intestine.

The cultural-dependent approach to the study of the fish gut microbiome used in this work did not provide us with an opportunity to determine the detailed microbial composition of the gut. Obviously, for fundamental studies of the microbiome and ecology of commensal microorganisms (formation and development processes, interaction with the host, comparison of taxonomic diversity, etc.), it is necessary to apply molecular genetic methods, in particular, 16S ribosomal sequencing and metagenomic analysis [105,106,107]. In doing so, this work showed that a culture-dependent approach revealed key shifts in the composition of gut microorganisms during the application of feed additives. Changes in the histomorphological parameters can reflect both the direct effect of feed ingredients on the intestinal mucosa and the gut response to changes in the microbial community. Studies of new feed formulations and feed additives using cultured microbiota and histological indices make it possible to assess their impact on the state of the GI tract. In our opinion, this approach avoids significant costs for the study while making it possible to identify patterns of influence of additives on the relative abundance of bacteria.

The results of the culture-dependent method used in this work are consistent with those obtained by other researchers using molecular genetic techniques. This method can find application in aquaculture, where there is a need to test many different feed formulations. The results of this study suggest the use of experimental model objects (e.g., *D. rerio*) to develop new feeds, especially to develop prebiotics and probiotics as immunity and microbiome modulators [45].

## 5. Conclusions

All of the investigated feed additives caused changes in the composition of the cultured microbiome of the anterior and posterior gut sections of *D. rerio*. The observed changes were predominantly found in the anterior part of the intestine. The most sensitive groups of microorganisms were *Bacillus* and *Serratia*. Lycopene had the most significant effect on the composition of the cultured microbiota (in all of the studied concentrations), leading to a significant increase in the relative occurrence of *Firmicutes* in the anterior part of the intestine. This culture-dependent method of studying the microbiome makes it possible to assess changes in some representatives of the main groups of microorganisms (*Firmicutes* and *Proteobacteria*). Despite the incompleteness of the data obtained by the culture-dependent method, its application makes it possible to assess the bioactive properties of the feed and feed additives and their impact on the microbiota involved in digestive processes. Studies of the histological structure of the anterior and posterior gut showed the relationship between the barrier and secretory functions of the gut and the composition of the microbiota while using butyric acid (1 and 2 g kg^−1^) and trace element chelated compounds (2 mg kg^−1^).

## Data Availability

Not applicable.

## Supplementary Materials

The following supporting information can be downloaded at: https://www.mdpi.com/article/10.3390/ani12182424/s1, Figure S1: Cultivated microbiota of the anterior and posterior sections of the intestine of the experimental groups of *D. rerio* receiving the studied supplements (*n* = 3). Unclassified bacterial groups are labeled within defined taxonomic groups: (a–f) relative occurrence of microbiota by bacterial class in the anterior and posterior gut sections, respectively. (a,b) Chelated compounds of trace elements; (b,c) butyric acid; (e,f) lycopene; Table S1: Phenotypical and biochemical properties of isolated bacteria. (+) more than 90% strains are positive; (−) more than 90% strains are negative; GN—Gram-negative; GP—Gram-positive; N—not done; V—variable results; Table S2: Morphometric parameters of the intestines of D. rerio receiving the studied feed additives; Table S3: Semi-quantitative data on the growth of bacterial cultures and their relative abundance.

## References

[1] Bharathi S., Antony C., Rajagopalasamy C. (2019). Functional Feed Additives Used in Fish Feeds. Int. J. Fish. Aquat. Stud..

[2] Ogunkalu O. (2019). Effects of Feed Additives in Fish Feed for Improvement of Aquaculture. Eurasian J. Food Sci. Technol..

[3] Dimitroglou A., Merrifield D.L., Carnevali O., Picchietti S., Avella M., Daniels C., Güroy D., Davies S.J. (2011). Microbial Manipulations to Improve Fish Health and Production—A Mediterranean Perspective. Fish Shellfish Immunol..

[4] Bulfon C., Volpatti D., Galeotti M. (2013). Current Research on the Use of Plant-Derived Products in Farmed Fish. Aquac. Res..

[5] Villasante A., Patro B., Chew B., Becerra M., Wacyk J., Overturf K., Powell M.S., Hardy R.W. (2015). Dietary Intake of Purple Corn Extract Reduces Fat Body Content and Improves Antioxidant Capacity and N-3 Polyunsaturated Fatty Acid Profile in Plasma of Rainbow Trout, *Oncorhynchus mykiss*. J. World Aquac. Soc..

[6] Steiner T. (2009). Phytogenics in Animal Nutrition: Natural Concepts to Optimize Gut Health and Performance.

[7] Lall S.P., Kaushik S.J. (2021). Nutrition and Metabolism of Minerals in Fish. Animals.

[8] Torno C., Staats S., Rimbach G., Schulz C. (2018). Effects of Resveratrol and Genistein on Nutrient Digestibility and Intestinal Histopathology of Rainbow Trout (*Oncorhynchus mykiss*). Aquaculture.

[9] Reda R.M., Selim K.M. (2014). Evaluation of *Bacillus amyloliquefaciens* on the Growth Performance, Intestinal Morphology, Hematology and Body Composition of *Nile tilapia*, *Oreochromis niloticus*. Aquac. Int..

[10] Guerreiro I., Couto A., Pérez-Jiménez A., Oliva-Teles A., Enes P. (2015). Gut Morphology and Hepatic Oxidative Status of European Sea Bass (*Dicentrarchus labrax*) Juveniles Fed Plant Feedstuffs or Fishmeal-Based Diets Supplemented with Short-Chain Fructo-Oligosaccharides and Xylo-Oligosaccharides. Br. J. Nutr..

[11] Brum A., Cardoso L., Chagas E.C., Chaves F.C.M., Mouriño J.L.P., Martins M.L. (2018). Histological Changes in *Nile tilapia* Fed Essential Oils of Clove Basil and Ginger after Challenge with *Streptococcus agalactiae*. Aquaculture.

[12] Nguyen C.D.H., Amoroso G., Ventura T., Elizur A. (2020). Assessing the Pyloric Caeca and Distal Gut Microbiota Correlation with Flesh Color in Atlantic Salmon (*Salmo salar* L., 1758). Microorganisms.

[13] Wang A.R., Ran C., Ringø E., Zhou Z.G. (2017). Progress in Fish Gastrointestinal Microbiota Research. Rev. Aquac..

[14] Hooper L.V., Littman D.R., Macpherson A.J. (2012). Interactions between the Microbiota and the Immune System. Science.

[15] Belkaid Y., Hand T.W. (2014). Role of the Microbiota in Immunity and Inflammation. Cell.

[16] Zarkasi K.Z., Taylor R.S., Abell G.C.J., Tamplin M.L., Glencross B.D., Bowman J.P. (2016). Atlantic Salmon (*Salmo salar* L.) Gastrointestinal Microbial Community Dynamics in Relation to Digesta Properties and Diet. Microb. Ecol..

[17] Petra A.I., Panagiotidou S., Hatziagelaki E., Stewart J.M., Conti P., Theoharides T.C. (2015). Gut-Microbiota-Brain Axis and Its Effect on Neuropsychiatric Disorders with Suspected Immune Dysregulation. Clin. Ther..

[18] Navarro-Barrón E., Hernández C., Llera-Herrera R., García-Gasca A., Gómez-Gil B. (2019). Overfeeding a High-Fat Diet Promotes Sex-Specific Alterations on the Gut Microbiota of the Zebrafish (*Danio rerio*). Zebrafish.

[19] Yang H.-T., Zou S.-S., Zhai L.-J., Wang Y., Zhang F.-M., An L.-G., Yang G.-W. (2017). Pathogen Invasion Changes the Intestinal Microbiota Composition and Induces Innate Immune Responses in the Zebrafish Intestine. Fish Shellfish Immunol..

[20] Castañeda-Monsalve V.A., Junca H., García-Bonilla E., Montoya-Campuzano O.I., Moreno-Herrera C.X. (2019). Characterization of the Gastrointestinal Bacterial Microbiome of Farmed Juvenile and Adult White Cachama (*Piaractus brachypomus*). Aquaculture.

[21] Sullam K.E., Essinger S.D., Lozupone C.A., O’connor M.P., Rosen G.L., Knight R., Kilham S.S., Russell J.A. (2012). Environmental and Ecological Factors That Shape the Gut Bacterial Communities of Fish: A Meta-Analysis. Mol. Ecol..

[22] Jin Y., Wu S., Zeng Z., Fu Z. (2017). Effects of Environmental Pollutants on Gut Microbiota. Environ. Pollut..

[23] Cantas L., Sørby J.R.T., Aleström P., Sørum H. (2012). Culturable Gut Microbiota Diversity in Zebrafish. Zebrafish.

[24] Hoseinifar S.H., Esteban M.Á., Cuesta A., Sun Y.-Z. (2015). Prebiotics and Fish Immune Response: A Review of Current Knowledge and Future Perspectives. Rev. Fish. Sci. Aquac..

[25] Martínez Cruz P., Ibáñez A.L., Monroy Hermosillo O.A., Ramírez Saad H.C. (2012). Use of Probiotics in Aquaculture. ISRN Microbiol..

[26] Bruce T.J., Neiger R.D., Brown M.L. (2018). Gut Histology, Immunology and the Intestinal Microbiota of Rainbow Trout, *Oncorhynchus mykiss*. Aquac. Res..

[27] González-Félix M.L., Gatlin D.M., Urquidez-Bejarano P., de la Reé-Rodríguez C., Duarte-Rodríguez L., Sánchez F., Casas-Reyes A., Yamamoto F.Y., Ochoa-Leyva A., Perez-Velazquez M. (2018). Effects of Commercial Dietary Prebiotic and Probiotic Supplements on Growth, Innate Immune Responses, and Intestinal Microbiota and Histology of *Totoaba macdonaldi*. Aquaculture.

[28] Wang Y., Abdullah, Zhong H., Wang J., Feng F. (2021). Dietary Glycerol Monolaurate Improved the Growth, Activity of Digestive Enzymes and Gut Microbiota in Zebrafish (*Danio rerio*). Aquac. Rep..

[29] Patula S., Wojno M., Pinnell L.J., Oliaro F., Cabay C., Molinari G.S., Kwasek K. (2021). Nutritional Programming with Dietary Soybean Meal and Its Effect on Gut Microbiota in Zebrafish (*Danio rerio*). Zebrafish.

[30] Reveco F.E., Øverland M., Romarheim O.H., Mydland L.T. (2014). Intestinal Bacterial Community Structure Differs between Healthy and Inflamed Intestines in Atlantic Salmon (*Salmo salar* L.). Aquaculture.

[31] Ingerslev H.-C., Strube M.L., von Gersdorff Jørgensen L., Dalsgaard I., Boye M., Madsen L. (2014). Diet Type Dictates the Gut Microbiota and the Immune Response against *Yersinia ruckeri* in Rainbow Trout (*Oncorhynchus mykiss*). Fish Shellfish Immunol..

[32] Ingerslev H.-C., von Gersdorff Jørgensen L., Lenz Strube M., Larsen N., Dalsgaard I., Boye M., Madsen L. (2014). The Development of the Gut Microbiota in Rainbow Trout (*Oncorhynchus mykiss*) Is Affected by First Feeding and Diet Type. Aquaculture.

[33] Arballo J., Amengual J., Erdman J.W. (2021). Lycopene: A Critical Review of Digestion, Absorption, Metabolism, and Excretion. Antioxidants.

[34] Lu Y., Zhou L., He S., Ren H.-L., Zhou N., Hu Z.-M. (2020). Lycopene Alleviates Disc Degeneration under Oxidative Stress through the Nrf2 Signaling Pathway. Mol. Cell. Probes.

[35] Gharibzahedi S.M.T., Jafari S.M. (2017). The Importance of Minerals in Human Nutrition: Bioavailability, Food Fortification, Processing Effects and Nanoencapsulation. Trends Food Sci. Technol..

[36] Walters M., Esfandi R., Tsopmo A. (2018). Potential of Food Hydrolyzed Proteins and Peptides to Chelate Iron or Calcium and Enhance Their Absorption. Foods.

[37] Zhang M., Wang Y., Zhao X., Liu C., Wang B., Zhou J. (2021). Mechanistic Basis and Preliminary Practice of Butyric Acid and Butyrate Sodium to Mitigate Gut Inflammatory Diseases: A Comprehensive Review. Nutr. Res..

[38] Ohira H., Tsutsui W., Fujioka Y. (2017). Are Short Chain Fatty Acids in Gut Microbiota Defensive Players for Inflammation and Atherosclerosis?. J. Atheroscler. Thromb..

[39] Grabowska M., Wawrzyniak D., Rolle K., Chomczyński P., Oziewicz S., Jurga S., Barciszewski J. (2019). Let Food Be Your Medicine: Nutraceutical properties of lycopene. Food Funct..

[40] Petyaev I.M. (2016). Lycopene Deficiency in Ageing and Cardiovascular Disease. Oxidative Med. Cell. Longev..

[41] Dawood M.A.O., Abdel-Tawwab M., Abdel-Latif H.M.R. (2020). Lycopene Reduces the Impacts of Aquatic Environmental Pollutants and Physical Stressors in Fish. Rev. Aquac..

[42] Seth A., Stemple D.L., Barroso I. (2013). The Emerging Use of Zebrafish to Model Metabolic Disease. Dis. Models Mech..

[43] López Nadal A., Ikeda-Ohtsubo W., Sipkema D., Peggs D., McGurk C., Forlenza M., Wiegertjes G.F., Brugman S. (2020). Feed, Microbiota, and Gut Immunity: Using the Zebrafish Model to Understand Fish Health. Front. Immunol..

[44] Oka T., Nishimura Y., Zang L., Hirano M., Shimada Y., Wang Z., Umemoto N., Kuroyanagi J., Nishimura N., Tanaka T. (2010). Diet-Induced Obesity in Zebrafish Shares Common Pathophysiological Pathways with Mammalian Obesity. BMC Physiol..

[45] Ulloa P.E. (2014). Zebrafish as Animal Model for Aquaculture Nutrition Research. Front. Genet..

[46] Roeselers G., Mittge E.K., Stephens W.Z., Parichy D.M., Cavanaugh C.M., Guillemin K., Rawls J.F. (2011). Evidence for a Core Gut Microbiota in the Zebrafish. ISME J..

[47] Oehlers S.H., Flores M.V., Chen T., Hall C.J., Crosier K.E., Crosier P.S. (2011). Topographical Distribution of Antimicrobial Genes in the Zebrafish Intestine. Dev. Comp. Immunol..

[48] Wallace K.N., Akhter S., Smith E.M., Lorent K., Pack M. (2005). Intestinal Growth and Differentiation in Zebrafish. Mech. Dev..

[49] Bischoff S.C., Volynets V. (2016). Nutritional Influences of Overfeeding on Experimental Outcomes in Laboratory Mice: Consequences for Gut Microbiota and Other Functional Studies. Int. J. Med. Microbiol..

[50] Alexander T.J.L. (1992). Methods of disease control. Disease of Swine.

[51] Nikiforov-Nikishin D.L., Nikiforov-Nikishin A.L., Bugaev O.G., Kochetkov N.I. (2021). Temperature Differentiation of Aquatic Microbiota of a Closed Water Supply System by the Example of Incubation of Microbiological Crops at 21 and 37 °C. IOP Conf. Ser. Earth Environ. Sci..

[52] Schreier H.J., Mirzoyan N., Saito K. (2010). Microbial diversity of biological filters in recirculating aquaculture systems. Curr. Opin. Biotechnol..

[53] Luca C., Ercolini D. (2008). Molecular Techniques in the Microbial Ecology of Fermented Foods.

[54] Cocolin L., Alessandria V., Dolci P., Gorra R., Rantsiou K. (2013). Culture Independent Methods to Assess the Diversity and Dynamics of Microbiota during Food Fermentation. Int. J. Food Microbiol..

[55] Jia S., Liu X., Huang Z., Li Y., Zhang L., Luo Y. (2018). Effects of Chitosan Oligosaccharides on Microbiota Composition of Silver Carp (*Hypophthalmichthys molitrix*) Determined by Culture-Dependent and Independent Methods during Chilled Storage. Int. J. Food Microbiol..

[56] Magne F., AbÃcly M., Boyer F., Morville P., Pochart P., Suau A. (2006). Low Species Diversity and High Interindividual Variability in Faeces of Preterm Infants as Revealed by Sequences of 16S RRNA Genes and PCR-Temporal Temperature Gradient Gel Electrophoresis Profiles. FEMS Microbiol. Ecol..

[57] Hiergeist A., Gläsner J., Reischl U., Gessner A. (2015). Analyses of Intestinal Microbiota: Culture versus Sequencing. ILAR J..

[58] Aleström P., D’Angelo L., Midtlyng P.J., Schorderet D.F., Schulte-Merker S., Sohm F., Warner S. (2019). Zebrafish: Housing and Husbandry Recommendations. Lab. Anim..

[59] Abdel-Latif H.M.R., Abdel-Tawwab M., Dawood M.A.O., Menanteau-Ledouble S., El-Matbouli M. (2020). Benefits of Dietary Butyric Acid, Sodium Butyrate, and Their Protected Forms in Aquafeeds: A Review. Rev. Fish. Sci. Aquac..

[60] Aalamifar H., Soltanian S., Vazirzadeh A., Akhlaghi M., Morshedi V., Gholamhosseini A., Torfi Mozanzadeh M. (2019). Dietary Butyric Acid Improved Growth, Digestive Enzyme Activities and Humoral Immune Parameters in Barramundi (*Lates calcarifer*). Aquac. Nutr..

[61] Gramm S., Sergey B., Nikita K., Viktor K., Alexei N.-N., Dmitry N.-N. (2020). Histological Changes in the Liver, Intestines and Kidneys of *Clarias gariepinus* When Using Feed with Chelated Compounds. Int. J. Pharm. Res..

[62] Nikiforov-Nikishin D., Kochetkov N., Klimov V., Bugaev O. (2022). Effects of Chelated Complexes and Probiotics on Histological and Morphometric Parameters of the Gastrointestinal Tract of Juvenile Carp (*Cyprinus carpio*). N. Z. J. Zool..

[63] Mézes M., Erdélyi M., Balogh K. (2012). Deposition of organic trace metal complexes as feed additives in farm animals. Eur. Chem. Bull..

[64] Langi P., Kiokias S., Varzakas T., Proestos C. (2018). Carotenoids: From Plants to Food and Feed Industries. Methods in Molecular Biology.

[65] Noakes D.E., Wallace L., Smith G.R. (1991). Bacterial Flora of the Uterus of Cows after Calving on Two Hygienically Contrasting Farms. Vet. Rec..

[66] Kim Suvarna S., Layton C., Bancroft J.D. (2019). Bancroft’s Theory and Practice of Histological Techniques.

[67] Menke A.L., Spitsbergen J.M., Wolterbeek A.P.M., Woutersen R.A. (2011). Normal Anatomy and Histology of the Adult Zebrafish. Toxicol. Pathol..

[68] Semova I., Carten J.D., Stombaugh J., Mackey L.C., Knight R., Farber S.A., Rawls J.F. (2012). Microbiota Regulate Intestinal Absorption and Metabolism of Fatty Acids in the Zebrafish. Cell Host Microbe.

[69] Falcinelli S., Rodiles A., Hatef A., Picchietti S., Cossignani L., Merrifield D.L., Unniappan S., Carnevali O. (2018). Influence of Probiotics Administration on Gut Microbiota Core. J. Clin. Gastroenterol..

[70] Antony Jesu Prabhu P., Schrama J.W., Mariojouls C., Godin S., Fontagné-Dicharry S., Geurden I., Surget A., Bouyssiere B., Kaushik S.J. (2014). Post-Prandial Changes in Plasma Mineral Levels in Rainbow Trout Fed a Complete Plant Ingredient Based Diet and the Effect of Supplemental Di-Calcium Phosphate. Aquaculture.

[71] Skrypnik K., Suliburska J. (2017). Association between the Gut Microbiota and Mineral Metabolism. J. Sci. Food Agric..

[72] Farzad R., Kuhn D.D., Smith S.A., O’Keefe S.F., Hines I.S., Bushman T.J., Galagarza O.A., Stevens A.M. (2021). Effects of Selenium-Enriched Prebiotic on the Growth Performance, Innate Immune Response, Oxidative Enzyme Activity and Microbiome of Rainbow Trout (*Oncorhynchus mykiss*). Aquaculture.

[73] Yausheva E., Miroshnikov S., Sizova E. (2018). Intestinal Microbiome of Broiler Chickens after Use of Nanoparticles and Metal Salts. Environ. Sci. Pollut. Res..

[74] Gillingham M.A.F., Borghesi F., Montero B.K., Migani F., Béchet A., Rendón-Martos M., Amat J.A., Dinelli E., Sommer S. (2021). Bioaccumulation of Trace Elements Affects Chick Body Condition and Gut Microbiome in Greater Flamingos. Sci. Total Environ..

[75] Stojković D., Kostić M., Smiljković M., Aleksić M., Vasiljević P., Nikolić M., Soković M. (2020). Linking Antimicrobial Potential of Natural Products Derived from Aquatic Organisms and Microbes Involved in Alzheimer’s Disease—A Review. Curr. Med. Chem..

[76] Vogt N.M., Kerby R.L., Dill-McFarland K.A., Harding S.J., Merluzzi A.P., Johnson S.C., Carlsson C.M., Asthana S., Zetterberg H., Blennow K. (2017). Gut Microbiome Alterations in Alzheimer’s Disease. Sci. Rep..

[77] Nie Y., Yang J., Zhou L., Yang Z., Liang J., Liu Y., Ma X., Qian Z., Hong P., Kalueff A.V. (2022). Marine Fungal Metabolite Butyrolactone I Prevents Cognitive Deficits by Relieving Inflammation and Intestinal Microbiota Imbalance on Aluminum Trichloride-Injured Zebrafish. J. Neuroinflamm..

[78] Patsiou D., del Rio-Cubilledo C., Catarino A.I., Summers S., Mohd Fahmi A., Boyle D., Fernandes T.F., Henry T.B. (2020). Exposure to Pb-Halide Perovskite Nanoparticles Can Deliver Bioavailable Pb but Does Not Alter Endogenous Gut Microbiota in Zebrafish. Sci. Total Environ..

[79] Zhou L., Limbu S.M., Shen Z., Zhai W., Qiao F., He A., Du Z., Zhang M. (2018). Environmental Concentrations of Antibiotics Impair Zebrafish Gut Health. Environ. Pollut..

[80] Xia H., Liu C., Li C.-C., Fu M., Takahashi S., Hu K.-Q., Aizawa K., Hiroyuki S., Wu G., Zhao L. (2018). Dietary Tomato Powder Inhibits High-Fat Diet–Promoted Hepatocellular Carcinoma with Alteration of Gut Microbiota in Mice Lacking Carotenoid Cleavage Enzymes. Cancer Prev. Res..

[81] Lin F., Wu H., Zeng M., Yu G., Dong S., Yang H. (2018). Probiotic/Prebiotic Correction for Adverse Effects of Iron Fortification on Intestinal Resistance to Salmonella Infection in Weaning Mice. Food Funct..

[82] Den Besten G., van Eunen K., Groen A.K., Venema K., Reijngoud D.-J., Bakker B.M. (2013). The Role of Short-Chain Fatty Acids in the Interplay between Diet, Gut Microbiota, and Host Energy Metabolism. J. Lipid Res..

[83] Azad M.A.K., Islam S.S., Sithi I.N., Ghosh A.K., Banu G.R., Bir J., Huq K.A. (2018). Effect of Probiotics on Immune Competence of Giant Freshwater Prawn *Macrobrachium rosenbergii*. Aquac. Res..

[84] Venkat H.K., Sahu N.P., Jain K.K. (2004). Effect of feeding Lactobacillus-based probiotics on the gut microbiota, growth and survival of postlarvae of *Macrobrachium rosenbergii* (de Man). Aquac. Res..

[85] Amoah K., Huang Q.C., Tan B.P., Zhang S., Chi S.Y., Yang Q.H., Liu H.Y., Dong X.H. (2019). Dietary supplementation of probiotic *Bacillus coagulans* ATCC 7050, improves the growth performance, intestinal morphology, microbiota, immune response, and disease confrontation of Pacific white shrimp, *Litopenaeus vannamei*. Fish Shellfish Immunol..

[86] Tao J., Wang S., Qiu H., Xie R., Zhang H., Chen N., Li S. (2022). Modulation of Growth Performance, Antioxidant Capacity, Non-Specific Immunity and Disease Resistance in Largemouth Bass (*Micropterus salmoides*) upon Compound Probiotic Cultures Inclusion. Fish Shellfish Immunol..

[87] López-Mondéjar R., Zühlke D., Becher D., Riedel K., Baldrian P. (2016). Cellulose and Hemicellulose Decomposition by Forest Soil Bacteria Proceeds by the Action of Structurally Variable Enzymatic Systems. Sci. Rep..

[88] Abid A., Davies S.J., Waines P., Emery M., Castex M., Gioacchini G., Carnevali O., Bickerdike R., Romero J., Merrifield D.L. (2013). Dietary synbiotic application modulates Atlantic salmon (*Salmo salar*) intestinal microbial communities and intestinal immunity. Fish Shellfish Immunol..

[89] Maslowski K.M., Mackay C.R. (2010). Diet, Gut Microbiota and Immune Responses. Nat. Immunol..

[90] Vidyasagar S., Barmeyer C., Geibel J., Binder H.J., Rajendran V.M. (2005). Role of Short-Chain Fatty Acids in Colonic HCO_3_ Secretion. Am. J. Physiol. Gastrointest. Liver Physiol..

[91] Annison G., Illman R.J., Topping D.L. (2003). Acetylated, Propionylated or Butyrylated Starches Raise Large Bowel Short-Chain Fatty Acids Preferentially When Fed to Rats. J. Nutr..

[92] Ward F.W., Coates M.E. (1987). Gastrointestinal PH Measurement in Rats: Influence of the Microbial Flora, Diet and Fasting. Lab. Anim..

[93] Hai N.V. (2015). The use of probiotics in aquaculture. J. Appl. Microbiol..

[94] Pelaseyed T., Bergström J.H., Gustafsson J.K., Ermund A., Birchenough G.M.H., Schütte A., van der Post S., Svensson F., Rodríguez-Piñeiro A.M., Nyström E.E.L. (2014). The Mucus and Mucins of the Goblet Cells and Enterocytes Provide the First Defense Line of the Gastrointestinal Tract and Interact with the Immune System. Immunol. Rev..

[95] Reboul E. (2019). Mechanisms of Carotenoid Intestinal Absorption: Where Do We Stand?. Nutrients.

[96] Lyu Y., Wu L., Wang F., Shen X., Lin D. (2018). Carotenoid Supplementation and Retinoic Acid in Immunoglobulin a Regulation of the Gut Microbiota Dysbiosis. Exp. Biol. Med..

[97] Lan C.-C., Love D.R. (2012). Molecular Characterisation of Bacterial Community Structure along the Intestinal Tract of Zebrafish (*Danio rerio*): A Pilot Study. ISRN Microbiol..

[98] Paul H.A., Bomhof M.R., Vogel H.J., Reimer R.A. (2016). Diet-Induced Changes in Maternal Gut Microbiota and Metabolomic Profiles Influence Programming of Offspring Obesity Risk in Rats. Sci. Rep..

[99] Arias-Jayo N., Abecia L., Alonso-Sáez L., Ramirez-Garcia A., Rodriguez A., Pardo M.A. (2018). High-Fat Diet Consumption Induces Microbiota Dysbiosis and Intestinal Inflammation in Zebrafish. Microb. Ecol..

[100] Rossi L.T., Sharpen A.R., Zimmermann J.A., Olivero C.R., Zbrun M.V., Frizzo L.S., Signorini M.L., Bacchetta C., Cian R.E., Cazenave J. (2020). Intestinal Microbiota Modulation in Juvenile Pacú (*Piaractus mesopotamicus*) by Supplementation with *Pyropia Columbina* and β-Carotene. Aquac. Int..

[101] Zhao H., Wang Y., Mu M., Guo M., Yu H., Xing M. (2020). Lycopene Alleviates Sulfamethoxazole-Induced Hepatotoxicity in Grass Carp (*Ctenopharyngodon idellus*) via Suppression of Oxidative Stress, Inflammation and Apoptosis. Food Funct..

[102] Reboul E. (2013). Absorption of Vitamin a and Carotenoids by the Enterocyte: Focus on Transport Proteins. Nutrients.

[103] Zhao B., Wu J., Li J., Bai Y., Luo Y., Ji B., Xia B., Liu Z., Tan X., Lv J. (2020). Lycopene Alleviates DSS-Induced Colitis and Behavioral Disorders via Mediating Microbes-Gut–Brain Axis Balance. J. Agric. Food Chem..

[104] Meng Y., Yang Y., Wang J. (2011). Effects of lycopene supplement on the antioxidant capacity of *Carassius auratus*. J. Anhui Agric. Univ..

[105] Qin J., Li R., Raes J., Arumugam M., Burgdorf K.S., Manichanh C., Nielsen T., Pons N., Levenez F., Yamada T. (2010). A Human Gut Microbial Gene Catalogue Established by Metagenomic Sequencing. Nature.

[106] Danilenko V.N., Yunes R.A., Ilyasov R.A., Kovtun A.S., Yanenko A.S., Kozlovsky Y.E., Sidorenko A.V., Kolomiets E.I. (2022). Research human and animal microbiome as a source of genetic and pharmacological resources for the development of innovative biotechnology in medicine, animal husbandry and agroindustrial complex. Adv. Mod. Biol..

[107] Danilenko V.N., Ilyasov R.A., Yunes R.A., Yanenko A.S., Kozlovsky Y.E., Sverchkova N.V., Kolomiets E.I. (2022). Animal microbiome: Search for biologically active ingredients for creation of probiotics and pharmabiotics. Adv. Mod. Biol..

