# Supplementary material for: Effects of Three Feed Additives on the Culturable Microbiota Composition and Histology of the Anterior and Posterior Intestines of Zebrafish (Danio rerio)
Source: Animals (Basel). 2022 Sep 14;12(18):2424. doi: 10.3390/ani12182424 (PMC9495144; doi:10.3390/ani12182424)
Supplement: Supplementary file 1 [file animals-12-02424-s001.zip › Supplementary.pdf]

Table S1. Phenotypical and biochemical properties of isolated bacteria. (+) more than 90% strains are positive; (-) more than 90% strains are negative; GN gram-negative; GP gram-positive; N not done; V variable results.

Gram stain and colony descriptions	Haemolysis	Motility	Catalase	Oxidase	Coagulase	Indole	Methyl-blue	Voges-Proskauer	Simmon Citrate
GN rods (round, smooth/roughened, gray, 2-3 mm columns)	V	+	+	-	-	+	+	-	+
GP cocci in pairs (round, smooth, gray, 2-3 mm colonies)	-	-	+	-	-	-	+	-	-
GN rods (round, flat, light green, 1-3 mm, colonies)	N	V	+	V	V	V	N	N	+
GN rods (red/white, rounded, 1-3 mm colonies)	N	+	+	+	N	-	V	+	+
GN cocci in pairs (white-gray, smooth, kidney-shaped, 2-3 mm colonies)	V	-	-	+	V	-	V	N	N
GP rods (large, white-gray, irregular, round/ kidney-shaped, 5-6 mm colonies)	+	+	+	V	N	-	N	+	+
GP rods (rounded, kidney-shaped, white, white-gray, even and uneven, 2-3/4-5 mm, colonies)	V	V	+	-	N	-	V	+	V
GN rods (rounded/narrow smooth, white/gray, 2-3/4 mm colonies)	V	V	V	V	-	V	+	-	V
GP cocci in pairs and chains (round, white, smooth, white, 1-3 mm colonies)	V	+	-	-	V	-	+	+	N
GN rods (large, smooth, white/gray, raised, 2-4 mm colonies)	V	+	+	V	-	-	+	-	V

Table S1. (Continued)

H2S production	Urea hydrolysis	Glucose	Lactose	Maltose	Mannitol	Esculin	Organisms
N	+	+	+	+	+	N	Acinetobacter
N	+	+	+	+	-	-	Staphylococcus
N	+	+	-	N	-	N	Unclassified_Cyanobacteria
-	-	+	-	+	+	N	Serratia
N	-	-	-	-	N	-	Veilonella
-	-	+	V	+	+	+	Bacillus
-	-	+	V	V	N	N	Unclassified_Firmicutes
N	-	V	V	V	+	N	Unclassified_Proteobacteria
+	-	+	-	V	N	-	Vagococcus
-	-	V	-	+	+	N	Unclassified_Enterobacteriaceae

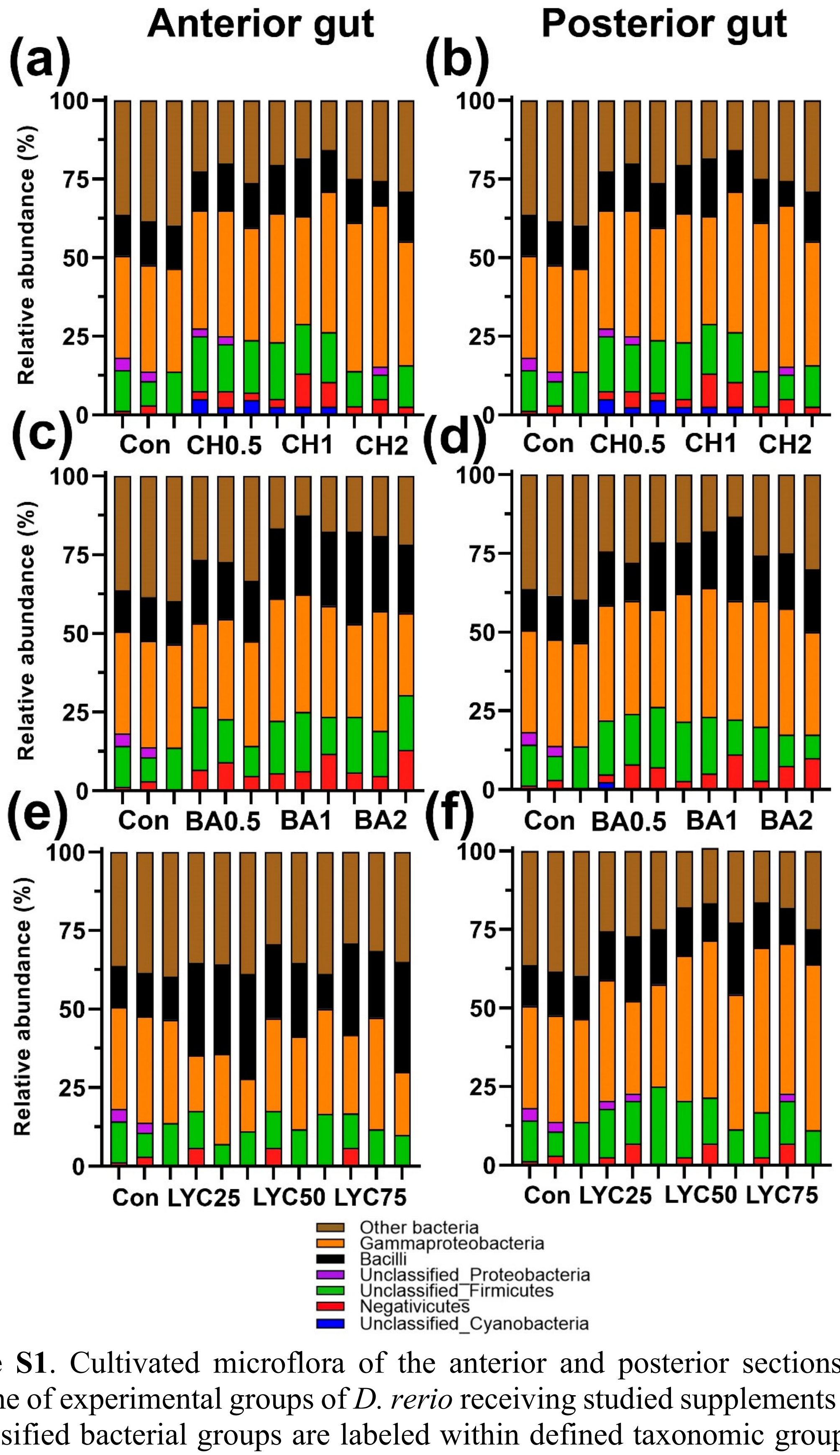

Figure S1. Cultivated microflora of the anterior and posterior sections of the intestine of experimental groups of *D. rerio* receiving studied supplements (n = 3). Unclassified bacterial groups are labeled within defined taxonomic groups: (a-f) relative occurrence of microbiota by bacterial class in the anterior and posterior gut sections, respectively. (**a, b**) chelated compounds of trace elements; (**b, c**) butyric acid; (**e, f**) lycopene.

Table S2. Morphometric parameters of the intestines of *D. rerio* receiving the studied feed additives. The value (* - p <0.05) from the Mann-Whitney test.

Experimental group	Hieght of the adsorbing epithelium	Width of lamina propria	Goblet cells area	Number of goblet cells per 100 µm of epithelium	Thickness of the muscularis
Con	27.16±1.4	4.29±0.06	109.89±7.18	1.65±0.3	11.08±0.66
CH0.5	28.43±1.28	4.18±0.36	94.87±7.29	1.73±0.1	10.28±0.93
CH1	27.56±3	4.73±0.36	100.65±9.36	1.55±0.46	15.52±3.91
CH2	29.34±2.96	5±0.33*	93.84±1.9	2.23±0.19	12.61±0.95
BA0.5	29.53±3.76	6.86±0.04*	148.03±12.95*	2.23±0.32	16.39±1.52*
BA1	27.92±0.84	5.18±0.66	126.38±11.13	1.98±0.18	12.28±1.52
BA2	23.94±2.93	7.55±0.93*	120.73±8.93	1.45±0.34	11.77±0.53
LYC25	24.7±1.25	7.23±0.32*	101.05±8.85	1.8±0.28	7.47±0.26
LYC50	26.49±1.01	4.94±0.74	101.05±6.39	1.98±0.34	11.12±0.66
LYC75	18.79±1.92*	5.14±0.44	83.77±11.24*	2.1±0.34	15.89±1.1*
